# Systemic Inflammation Is Associated with Resting Premature Atrial and Ventricular Contractions in American Older Adults

**DOI:** 10.19102/icrm.2026.17056

**Published:** 2026-05-15

**Authors:** Laith Ashour, Mohammad Obar, Afnan Yaser, Qusai Aref Fraihat, Batool Abuqudaira, Nour Ebeidat, Lara Al-Ghwiery, Mohammad Zaita, Enas Subbah, Abdelrahman Kaoukji, Lujain Badran

**Affiliations:** 1 Public Health Institute, Liverpool John Moores University, Liverpool, UK; 2 Jordan University Hospital, The University of Jordan, Amman, Jordan; 3 Faculty of Medicine, Al-Balqa Applied University, Al-Salt, Jordan; 4 Al Hussain New Salt Hospital, Al-Salt, Jordan; 5 Prince Hamza Hospital, Amman, Jordan; 6 Specialty Hospital, Private Sector, Amman, Jordan; 7 Al Essra Hospital, Private Sector, Amman, Jordan

**Keywords:** Older adults, premature atrial contraction, premature ventricular contraction, systemic inflammation

## Abstract

Prior studies have demonstrated links between systemic inflammation and cardiac arrhythmias. However, evidence on the association between inflammatory biomarkers and resting premature atrial contractions (PACs) or premature ventricular contractions (PVCs) in a national sample of older adults remains limited. Using biomarker data from the Midlife in the United States (MIDUS 3) study (2017–2022), we conducted non-parametric univariate analyses to assess associations between inflammatory markers—specifically, interleukin (IL)-6, IL-8, IL-10, tumor necrosis factor-α (TNF)-α, C-reactive protein (CRP), and fibrinogen—and baseline electrocardiographic (ECG) findings categorized as normal, PVCs, or PACs. We conducted a subsequent multivariate analysis of covariance adjusted for age, waist–hip ratio (WHR), and creatinine, the only confounders showing significant associations. A total of 699 participants were included, 395 (57%) of whom were women, with a mean age of 65.7 years (standard deviation, 9.6 years). The Kruskal–Wallis tests demonstrated significant associations of ECG pattern with IL-6 (median, normal 0.98 vs. PVCs 1.06 vs. PACs 1.40; *P* = .006) and IL-8 (12.4 vs. 13.99 vs. 13.13; *P* = .028). No significant associations were found for IL-10, TNF-α, CRP, or fibrinogen (*P* > .05). After adjustment for age, WHR, and creatinine, the ECG pattern remained significantly associated with IL-6 and IL-8 (*P* = .034). Elevated IL-6 and IL-8 levels are associated with resting PVCs and PACs in older adults. WHR and renal function also represent significant related factors that warrant consideration in future pathophysiological research.

## Introduction

Premature atrial contractions (PACs) and premature ventricular contractions (PVCs) are among the most commonly encountered arrhythmias in older adults, with multiple studies demonstrating age-related increases in their prevalence.^1–3^ Reported rates vary widely, largely depending on the duration and method of electrocardiographic (ECG) monitoring. For example, the Cardiovascular Health Study identified PACs in 4.2% and PVCs in 5.2% of elderly participants using standard 10-s, 12-lead resting ECGs.^4^ In contrast, studies employing extended monitoring have shown substantially higher detection rates. Among individuals aged >50 years, 24-h Holter monitoring detected at least one PAC in 99% of participants, with the median PAC frequency rising from 0.8/h at age 50 years to 6.5/h at age 70 years.^5^ Additional evidence demonstrates that ectopic burden—defined as the number of premature beats in 24 h—increases with age for both PACs and PVCs.^6,7^

Several etiologic factors have been proposed to contribute to PACs and PVCs, though the precise mechanisms remain incompletely understood. These factors include electrolyte imbalances, structural heart disease, prior myocardial infarction, medication effects, infectious processes, and additional systemic or cardiac stressors.^8,9^ Over time, frequent ectopy may result in structural cardiac disturbances, such as reversible PVC-induced cardiomyopathy, which can present similarly to heart failure.^10^ The risk of such complications is compounded when PVCs and PACs coexist.^11^

Older adults commonly experience metabolic abnormalities—such as obesity, insulin resistance, diabetes, and dyslipidemia—that promote chronic low-grade inflammation. Circulating cytokine levels tend to be elevated in these conditions, and this sustained inflammatory state may increase susceptibility to arrhythmogenesis.^12–15^ Growing evidence indicates that systemic inflammation alters cardiac electrophysiology, increasing the likelihood of premature atrial or ventricular activity. Among inflammatory mediators, interleukin-6 (IL-6) consistently emerges as a key cytokine implicated in arrhythmia-related electrical remodeling. IL-6 and related cytokines may contribute to fibrotic or structural changes that predispose individuals to PACs and PVCs.^16,17^

Given the previous findings linking inflammatory markers to abnormal electrical remodeling in patient populations with overt systemic disease, evaluating these associations in the general elderly population is important, particularly considering the high prevalence of ECG abnormalities in this age group. Clarifying whether inflammation contributes to resting PACs and PVCs may also improve understanding of their pathophysiology and inform risk stratification. We hypothesized that systemic inflammatory biomarkers are associated with resting PACs and PVCs in older adults.

## Materials and methods

### Study population

The Midlife in the United States (MIDUS) study is a longitudinal research project that began in 1995 and administered national surveys to >7000 Americans aged 25–75 years on the general health of the population, with oversampling from five metropolitan cities. This study aimed to assess the role of psychological, behavioral, and social factors in understanding age-related physical and mental health. Follow-up MIDUS studies were conducted over the next years, with MIDUS 2 conducted in 2009 and MIDUS 3 carried out in 2013. Both studies involved biomarker data collection. For MIDUS 3, from which we derived the data, the biomarker data project commenced in 2017. Data were collected during a 24-h stay visit from participants invited to one of three research units (University of California, Los Angeles; University of Wisconsin–Madison; and Georgetown University). The data collected assess the functionality of musculoskeletal, neurological, immune, and other systems. The data-collection protocol included trained clinicians and staff who collected specimens needed for biomarker data on-site. The pooled sample of the MIDUS 3 Biomarker Project comprised 747 invited participants (longitudinal survey sample [n = 644] and a Milwaukee sample [n = 103]), with an overall adjusted response rate of 64.3% (747/1162).

In the current study, participants with either normal, PVC, or PAC ECG patterns were included in the analysis. All other cases were excluded. This resulted in a pooled sample of 699 participants.

### Study design

The variables that were gathered and analyzed in this cross-sectional research were exclusively derived from the MIDUS 3 2017–2022 biomarker dataset.

### Study variables

#### Electrocardiographic measures

The resting ECG patterns were measured in the MIDUS 3 Biomarker Project via a detailed approach. In MIDUS 3, for ECG, measurement was done via electrodes placed on the right and left shoulders and in the left lower quadrant. Resting (baseline) ECG readings were recorded for the patient after 11 min (660 s; two epochs) of sitting quietly, and the patient was advised to remain silent during the procedure. The physiology of the ECG signal was determined based on visual review. If the physiology did not contain any arrhythmias identifiable by visual scan, the baseline physiology was coded as “normal.” Similarly, PVCs and PACs were identified visually by trained medical staff. Further details can be found in the documentation of the psychophysiology protocol of the MIDUS 3 Biomarker Project.^18^

#### Inflammatory biomarkers

IL-6, IL-8, IL-10, and tumor necrosis factor (TNF)-α were assayed in the MIDUS Biocore Laboratory using electrochemiluminescence (V-PLEX Plus Proinflammatory Panel 1 Human Kit; Meso Scale Discovery, Rockville, MD, USA). This platform uses multispot 96-well plates coated with cytokine-specific capture antibodies. Following sample incubation, Sulfo-tagged detection antibodies were applied, and signal intensity was quantified via the MESO QuickPlex SQ120 imager (Meso Scale Discovery). This instrument measures the intensity of the emitted light to provide a quantitative measure of analytes in each sample.^19^ Fibrinogen and C-reactive protein (CRP) quantification was conducted at the Laboratory for Clinical Biochemistry Research (University of Vermont, Burlington, VT, USA). Fibrinogen was measured using BN™ II nephelometry (Siemens Healthineers, Erlangen, Germany), while CRP was measured via electrochemiluminescence (V-PLEX Plus Human CRP Kit) on a Meso Scale Discovery platform using frozen serum (E. Cornell, Laboratory for Clinical Biochemistry Research, University of Vermont, personal communication, January 20, 2023).

#### Metabolic covariates

To identify potential confounders, univariate analyses also included creatinine, hemoglobin A1c, lipid indices (low-density lipoprotein, high-density lipoprotein, total cholesterol, triglycerides), anthropometric measures (body mass index [BMI] and waist–hip ratio [WHR]), fasting insulin-like growth factor-1, systolic and diastolic blood pressures (mean of the second and third readings), and the MIDUS Healthy Eating Index (MIDUS-HEI). The HEI score (0–11 points) reflects the frequency of healthy versus unhealthy food intake, with higher scores indicating healthier dietary patterns. See MIDUS 3 Biomarker Project documentation of blood, urine, and saliva data for more details on lipids and other indices’ measurement assays and procedures.^18^

### Statistical analysis

Descriptive statistics were used to characterize the sample. Means and standard deviations (SDs) were reported for normally distributed continuous variables, while medians and interquartile ranges were used for non-normally distributed variables. Categorical data were reported as counts and percentages.

Given the non-normal distributions of biomarkers (based on residual Q–Q plots), univariate associations between inflammatory markers and ECG categories were examined using the Kruskal–Wallis test with Dunn’s post hoc comparisons. The Kruskal–Wallis test or one-way analysis of variance was used to assess associations between ECG categories and metabolic variables.

A multivariate analysis of covariance (MANCOVA) model was applied to examine associations between ECG pattern and the inflammatory markers that were significant in univariate tests, adjusting for significant confounders (WHR and creatinine). Controlled variables were log-transformed to satisfy model assumptions. All analytic assumptions were met. Missing data were handled using list-wise deletion because the data were not missing at random.

Graphical displays included violin and box plots. Statistical significance was defined as *P* < .05. Analyses were performed using Jamovi software (version 2.6.13; The Jamovi Project, Sydney, Australia).

### Ethical approval

This study involved secondary analysis of publicly available MIDUS data. All MIDUS protocols were approved by the University of Wisconsin–Madison Institutional Review Board.

## Results

### Basic features

The total number of valid cases (participants) in our study was 699, with 395 (57%) being women, 297 (42%) having a history of regular smoking, and the mean age being 65.7 years (SD, 9.6 years). Basic features are displayed in **Table 1**.

**Table 1:** Basic Features of the Participants

	Count	% of Total
Sex		
Male	304	43%
Female	395	57%
History of regular smoking		
Yes	297	42%
No	402	58%
Baseline ECG pattern		
Normal	471	67%
PVCs	144	21%
PACs	84	12%
Total	699	100%

*Abbreviations:* ECG, electrocardiographic; PAC, premature atrial contraction; PVC, premature ventricular contraction.

The basic metabolic features of the study participants are displayed in **Table 2**. As can be seen, the results indicate that the metabolic features of older adults are not correlated with baseline ECG patterns, except for the WHR (*P* = .023) and creatinine levels (*P* = .007). Of note, among basic non-metabolic features, age was significantly associated with baseline ECG pattern (*P* < .001).

**Table 2:** Association Between Metabolic Factors and Baseline ECG Patterns in General American Older Adults Population

	Normal (n = 459)	PVCs (n = 137)	PACs (n = 83)	Total (n = 679)	*P* Value	Test*
MIDUS-HEI					.817	2
Median (IQR)	5.5 (4.5–6.5)	5.5 (4.5–6.5)	5.5 (4.2–6.5)	5.5 (4.5–6.5)		
Creatinine, mg/dL					**.007**	2
Median (IQR)	0.9 (0.8–1.0)	0.9 (0.8–1.1)	0.9 (0.8–1.1)	0.9 (0.8–1.0)		
Fasting IGF-1, ng/mL					.175	1
Mean (SD)	135.4 (45.8)	136.4 (36.4)	126.3 (38.0)	134.5 (43.2)		
HbA1c					.545	2
Median (IQR)	5.5 (5.3–5.9)	5.5 (5.3–6.0)	5.6 (5.3–6.0)	5.6 (5.3–5.9)		
Cholesterol, mg/dL					.166	1
Mean (SD)	180.4 (39.4)	176.7 (38.1)	172.1 (37.8)	178.6 (39.0)		
Triglycerides, mg/dL					.881	2
Median (IQR)	98.0 (71.0–140.5)	95.0 (72.0–130.0)	97.0 (72.5–124.5)	97.0 (72.0–135.0)		
HDL, mg/dL					.386	2
Median (IQR)	51.0 (41.5–63.0)	55.0 (44.0–66.0)	52.0 (42.0–63.5)	52.0 (42.0–64.0)		
LDL, mg/dL					.187	1
Mean (SD)	103.6 (34.6)	99.3 (32.0)	97.7 (32.0)	102.0 (33.8)		
BMI, kg/m^2^					.379	2
Median (IQR)	29.1 (25.4–33.2)	28.1 (25.2–32.3)	27.7 (24.7–32.9)	28.8 (25.2–33.0)		
WHR, ratio					**.023**	1
Mean (SD)	0.90 (0.28)	0.94 (0.22)	0.96 (0.27)	0.9 (0.1)		
SBP, mmHg					.648	2
Median (IQR)	126.0 (116.0–137.0)	128.0 (116.0–139.0)	129.0 (117.5–141.0)	126.0 (116.0–138.0)		
DBP, mmHg					.248	1
Mean (SD)	77.7 (9.3)	76.2 (9.1)	77.2 (9.3)	77.4 (9.3)		

*Abbreviations:* ANOVA, analysis of variance; BMI, body mass index; DBP, diastolic blood pressure; HbA1c, hemoglobin A1c; HDL, high-density lipoprotein; IGF-1, insulin-like growth factor-1; IQR, interquartile range; LDL, low-density lipoprotein; MIDUS-HEI, Midlife in the United States Healthy Eating Index; PAC, premature atrial contraction; PVC, premature ventricular contraction; SBP, systolic blood pressure; SD, standard deviation; WHR, waist–hip ratio. *Tests: 1. One-way ANOVA. 2. Kruskal–Wallis test. Bold *P* values indicate statistical significance.

### Univariate difference in serum biomarkers levels across electrocardiographic patterns

After confirming non-normal distributions for the inflammatory biomarkers, the Kruskal–Wallis test was applied. Significant differences across ECG categories were observed for IL-6 (*P* = .006) and IL-8 (*P* = .028). The median values for each ECG group are presented in **Table 3**. No other inflammatory markers demonstrated significant variation across ECG patterns.

**Table 3:** Comparative Assessment of Serum Inflammatory Biomarkers According to Resting Electrocardiographic Patterns

Kruskal–Wallis Test Results	Median (25^th^ and 75^th^ Percentiles)									
	Normal			PVCs			PACs			*P* Value
Blood serum MSD IL-6, pg/mL	0.98	0.64	1.56	1.06	0.71	1.6	1.4	0.8	1.91	.006
Blood serum MSD IL-8, pg/mL	12.4	9.18	16.08	13.99	10.09	18	13.13	9.66	17.29	**.028**
Blood serum MSD IL-10, pg/mL	0.24	0.17	0.34	0.23	0.17	0.32	0.26	0.18	0.4	.192
Blood serum MSD TNF-α, pg/mL	2.08	1.75	2.57	2.17	1.75	2.6	2.3	1.77	2.95	.116
C-reactive protein, μg/mL	1.72	0.76	4.55	1.65	0.62	3.7	1.8	0.87	5.31	.437
Blood fibrinogen, mg/dL	364	319	417	370.5	319	434.75	371	328.75	433.25	.235

*Abbreviations:* IL, interleukin; MSD, Meso Scale Discovery; PAC, premature atrial contraction; PVC, premature ventricular contraction; TNF-α, tumor necrosis factor-α. Bold *P* values indicate statistical significance.

Dunn’s post hoc pairwise comparisons showed that IL-6 levels differed significantly between normal and PAC patterns (*P* < .001) and between PVC and PAC patterns (*P* = .016), while no significant difference was detected between the normal and PVC groups (*P* = .478). For IL-8, post hoc analyses indicated a significant difference only between normal and PVC patterns (*P* = .013). Comparisons between normal and PACs (*P* = .194) and between PVCs and PACs (*P* = .545) were not significant.

**Figure 1:**
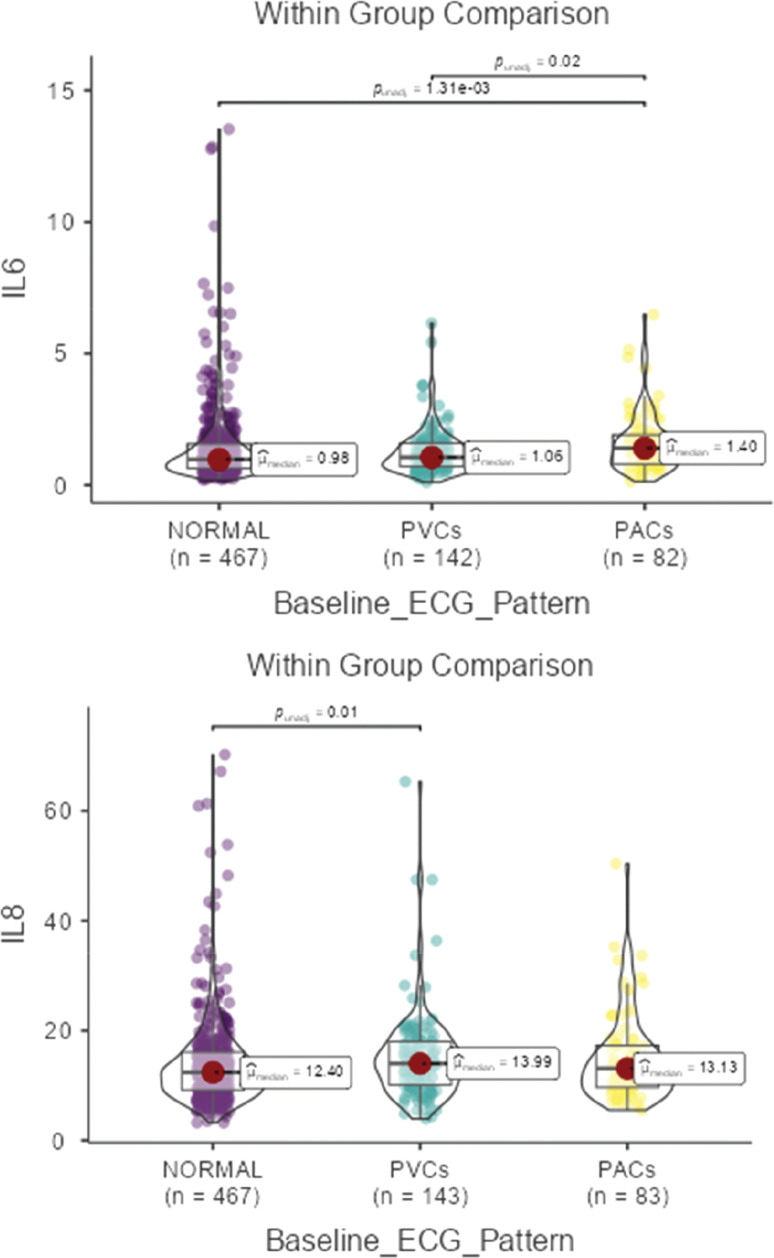
Pairwise comparison of inflammatory biomarkers levels across different electrocardiographic patterns in American older adults. *Abbreviations:* IL, interleukin; PAC, premature atrial contraction; PVC, premature ventricular contraction.

To satisfy model assumptions, creatinine, IL-6, and IL-8 values were log-transformed prior to multivariate analysis. A MANCOVA was then performed to evaluate the association between baseline ECG pattern and IL-6 and IL-8, adjusting for age and the two metabolic covariates identified as significant in univariate analyses (creatinine and WHR; see **Table 2**). The MANCOVA revealed a significant multivariate association between ECG category and IL-6 and IL-8 levels, respectively (*P* = .034). Follow-up univariate models demonstrated that IL-6 was significantly associated with ECG pattern (*P* = .024), WHR (*P* < .001), and creatinine (*P* < .001). In contrast, none of these factors were significantly associated with IL-8 (all *P* > .05). **Figure 1** presents the graphical representations of these distributions.

## Discussion

In this study, we identified a significant association between elevated IL-6 and IL-8 concentrations and resting atrial and ventricular ectopy in older adults. While the absolute concentrations are in the normal range, the approximately 40% higher median IL-6 levels observed in the PAC group and the 13% higher median IL-8 levels observed in the PVC group suggest a relative shift in inflammatory tone that may be biologically relevant in the context of arrhythmogenesis. These results suggest that systemic inflammation—particularly the IL-6–mediated inflammatory milieu common in older populations—is associated with PACs and PVCs at rest. Although no prior research has specifically examined this association in a general community-dwelling elderly cohort, IL-6 has been strongly linked to other age-related or disease-related arrhythmias. Elevated IL-6 levels, and especially the IL-6-174CC genotype associated with increased IL-6 expression, have been shown to predict atrial fibrillation (AF) in elderly patients with coronary disease, whereas other markers such as CRP and TNF-α do not demonstrate similar associations.^20^ IL-6 trans-signaling has also been implicated in AF pathogenesis through its role in promoting atrial inflammation, fibrosis, and conduction abnormalities, and selective blockade of this pathway has been shown experimentally to reduce AF susceptibility and attenuate structural remodeling.^21^ Beyond electrophysiologic remodeling, elevated IL-6 in AF patients independently predicts stroke and mortality risk, and the incorporation of IL-6 into the CHA₂DS₂-VASc score has been proposed to improve risk stratification.^22^ IL-6 also promotes cardiac sympathetic hyperactivity and left stellate ganglion remodeling after myocardial infarction, increasing susceptibility to ventricular arrhythmias,^23^ and has been identified as a novel risk factor for QT prolongation and torsades de pointes in at-risk individuals.^24^ Collectively, these findings highlight IL-6 as a central mediator across diverse arrhythmic pathways and align with our observations regarding PACs and PVCs.

IL-8 also demonstrated significant associations with resting ectopy in our population. Participants with PVCs had the highest IL-8 concentrations, followed by those with PACs. Prior studies similarly implicate IL-8 in arrhythmogenesis. Elevated IL-8 levels have been observed in individuals with paroxysmal AF and myocardial inflammation, and levels may remain persistently elevated even after restoration of sinus rhythm.^25^ Preoperative IL-8 concentrations have also predicted postoperative PVCs and PACs following coronary artery bypass grafting (CABG), suggesting that heightened inflammatory tone predisposes to both atrial and ventricular ectopy.^26^ Our findings extend this evidence to a national sample of community-dwelling older adults, reinforcing IL-8–mediated pathways as potential contributors to arrhythmic vulnerability and suggesting that IL-8 may serve as a biomarker of arrhythmic risk alongside IL-6. The other inflammatory marker (ie, CRP) had significant association with PVCs in prior studies.^26^ However, the difference in sample characteristics (community-dwelling older adults vs. CABG patients) can explain this discrepancy. It is well known that patients with coronary artery disease have higher baseline CRP levels, which explains this difference in the results.

The pathophysiological basis for these associations likely reflects several converging mechanisms. IL-6 activates the IL-6 receptor/Janus kinase–signal transducer and activator of transcription signaling pathway, which suppresses the *hERG*-mediated rapid delayed rectifier potassium current (I_Kr_), prolongs the cardiac action potential, and increases susceptibility to early after-depolarizations.^27,28^ In parallel, IL-6 contributes to structural remodeling by downregulating cardiac connexins, thereby impairing electrical coupling and increasing arrhythmic vulnerability.^29^ IL-6 and IL-8 also promote neutrophil recruitment and activation as part of the systemic inflammatory response, leading to excessive generation of reactive oxygen species (ROS).^30^ Elevated ROS disrupt ionic homeostasis, alter Nav1.5 channel kinetics, impair Ca^2+^ handling, induce mitochondrial dysfunction, and promote gap-junction remodeling—all key drivers of arrhythmogenesis.^31^ These molecular pathways provide biologically plausible mechanisms linking IL-6 and IL-8 to the increased burden of resting PACs and PVCs observed in our cohort.

In contrast, IL-10, TNF-α, and CRP were not significantly associated with resting PACs or PVCs in our analyses. This may reflect the characteristics of our study population, which included older adults with high BMIs and possible medication use—factors that may obscure inflammatory signals. For example, CRP levels may be attenuated among statin users, and individuals with limited physical activity may demonstrate persistently elevated inflammatory risk profiles despite biomarker variability.^32^ Moreover, several studies have similarly reported no association between CRP, TNF-α, or IL-10 and specific arrhythmias. CRP has shown no relationship with abnormal QT prolongation^33^ and does not appear to independently increase the risk of AF or related arrhythmias.^34,35^ In a multivariable analysis among patients with coronary artery disease, TNF-α and high-sensitivity CRP were not associated with AF after adjusting for other biomarkers, whereas IL-6 remained the only significant inflammatory predictor.^36^ Similarly, a cohort of postmenopausal women demonstrated no association between TNF-α or IL-10 and incident AF.^37^ Although these studies focused on arrhythmias other than resting ectopy, they collectively suggest that not all inflammatory biomarkers contribute equally to arrhythmogenesis, supporting our findings.

In terms of participant characteristics, WHR showed a strong association with both IL-6 levels and ECG pattern. At the population level, obesity—including elevated WHR—has been associated with incident AF, as demonstrated in a Swedish cohort of individuals in their late 50s.^38^ Mechanistically, expanding adipose tissue outgrows its blood supply, leading to local hypoxia, macrophage recruitment, and increased IL-6 secretion.^39^ Hypoxia also activates the nuclear factor κB pathway, further inducing cytokine production.^40^ These cytokines contribute to chronic low-grade inflammation, a central mechanism underlying obesity-related comorbidities.^41^ IL-6 additionally promotes cardiac fibroblast proliferation and collagen synthesis, accelerating interstitial fibrosis,^42^ a recognized risk factor for arrhythmogenesis.^43^ Thus, the association between WHR and resting ectopy in our cohort is biologically plausible and may reflect adiposity-driven IL-6 elevation, although this mechanism warrants further investigation.

Creatinine levels were significantly associated with baseline ECG patterns, with higher creatinine levels observed in individuals exhibiting resting PVCs or PACs. Although creatinine itself did not show a significant association with these two arrhythmias in the literature, previous research supports a comparable link between renal dysfunction and cardiac rhythm abnormalities.

At the molecular and autonomic levels, mounting evidence links chronic kidney disease (CKD) to electrophysiologic disturbances. Mechanisms contributing to this association include heightened sympathetic activation, chronic stimulation of the renin–angiotensin–aldosterone system, and accumulation of uremic toxins such as indoxyl sulfate and fibroblast growth factor 23, all of which can promote arrhythmogenesis.^44^ Collectively, the physiologic and metabolic derangements observed in CKD facilitate the early onset and progression of cardiac arrhythmias.^45^ Meta-analytic evidence also indicates that CKD increases the risk of incident AF by approximately 47%.^46^ These findings support our observations; however, our results demonstrate that minor increases in creatinine may be associated with arrhythmias—specifically PVCs and PACs—even at rest, underscoring the need to account for renal status in the pathophysiology of resting ectopy.

### Implications

Our findings emphasize the relevance of inflammatory biomarkers in the evaluation and management of resting PVCs and PACs in older adults. They also highlight the importance of addressing modifiable risk factors—such as obesity and impaired renal function—in preventing these arrhythmias. Lifestyle interventions, particularly increased physical activity, remain foundational in mitigating these risks. Other weight-reduction strategies have similarly demonstrated benefit; for instance, among 81 US patients undergoing bariatric surgery, postoperative reductions in pericardial fat thickness were significantly associated with shorter corrected QT intervals and improvements in ventricular conduction parameters.^47^

Renal dysfunction should also be recognized as a meaningful contributor to arrhythmic risk. Pharmacologic interventions, including sodium–glucose cotransporter 2 inhibitors, possess demonstrated anti-arrhythmogenic effects in both diabetic and non-diabetic populations.^44^ Additionally, IL-6 represents a promising therapeutic target for arrhythmias associated with inflammatory states,^48^ although its role in the pathophysiology of PVCs and PACs specifically requires further study.

## Conclusion

Our study identifies novel associations between circulating inflammatory markers and resting PVCs and PACs in older adults. Moreover, metabolic features—particularly renal impairment and increased WHR—appear to play important roles in the pathophysiology of these arrhythmias. Continued research is warranted to elucidate the molecular mechanisms underlying these findings and to explore targeted therapeutic strategies for this population.

## Data Availability

Data analyzed in this work are publicly available at the MIDUS 3 Biomarker Project webpage (https://www.icpsr.umich.edu/web/NACDA/studies/38837).

## References

[1] Dong Y, Li X, Zheng W (2022). Prevalence and heart rate variability characteristics of premature ventricular contractions detected by 24-hour Holter among outpatients with palpitations in China: a cross-sectional study. BMJ Open.

[2] Mannina C, Jin Z, Matsumoto K (2021). Frequency of cardiac arrhythmias in older adults: findings from the Subclinical Atrial Fibrillation and Risk of Ischemic Stroke (SAFARIS) study. Int J Cardiol.

[3] Torrado J, Sima A, Comstuck C (2025). Prevalence of frequent premature ventricular contractions and left-ventricular systolic dysfunction in patients receiving Holter monitoring. J Cardiovasc Electrophysiol.

[4] Nguyen KT, Vittinghoff E, Dewland TA (2017). Ectopy on a single 12-lead ECG, incident cardiac myopathy, and death in the community. J Am Heart Assoc.

[5] Conen D, Adam M, Roche F (2012). Premature atrial contractions in the general population: frequency and risk factors. Circulation.

[6] Huang TC, Lee PT, Huang MS, Su PF, Liu PY (2021). Higher premature atrial complex burden from the Holter examination predicts poor cardiovascular outcome. Sci Rep.

[7] Krumerman A, Di Biase L, Gerstenfeld E (2024). Premature ventricular complexes: assessing burden density in a large national cohort to better define optimal ECG monitoring duration. Heart Rhythm.

[8] Heaton J, Yandrapalli S (2025). StatPearls.

[9] Sattar Y, Hashmi MF (2025). StatPearls.

[10] Shen X, Zhu X, Zuo L, Liu X, Qin M (2023). Mechanisms and risk factors for premature ventricular contraction induced cardiomyopathy. Rev Cardiovasc Med.

[11] Måneheim A, Engström G, Juhlin T, Persson A, Zaigham S, Johnson LSB (2022). Elevated premature ventricular complex counts on 24-hour electrocardiogram predict incident atrial fibrillation and heart failure – a prospective population-based cohort study. Heart Rhythm O2.

[12] Wang Z, Jiao S, Chen J (2023). The relationship between frequent premature ventricular complexes and epicardial adipose tissue volume. Front Endocrinol (Lausanne).

[13] Dobrev D, Heijman J, Hiram R, Li N, Nattel S (2023). Inflammatory signalling in atrial cardiomyocytes: a novel unifying principle in atrial fibrillation pathophysiology. Nat Rev Cardiol.

[14] Spinelli V, Laurino A, Balducci V (2024). Interleukin-6 modulates the expression and function of HCN channels: a link between inflammation and atrial electrogenesis. Int J Mol Sci.

[15] Patel KHK, Reddy RK, Sau A, Sivanandarajah P, Ardissino M, Ng FS (2022). Obesity as a risk factor for cardiac arrhythmias. BMJ Med.

[16] Chen Y, Wu S, Li W (2018). Higher high-sensitivity C reactive protein is associated with future premature ventricular contraction: a community based prospective cohort study. Sci Rep.

[17] Ghadrdoost B, Aboutaleb N, Nikougoftar Zarif M, Nakhlestani M, Haghjoo M, Sameie S (2019). Association between cytokines and two circulating micro-RNAs and development of premature ventricular contractions-induced cardiomyopathy. Iran J Basic Med Sci.

[18] Ryff CD, Seema TE, Weinstein M (2023). Midlife in the United States (MIDUS 3): Biomarker Project, 2017–2022 (ICPSR 38837).

[19] Meso Scale Diagnostics, LLC (2020). MSD Cytokine Assays Proinflammatory Panel 1 (human) Kits [package insert].

[20] Marcus GM, Whooley MA, Glidden DV, Pawlikowska L, Zaroff JG, Olgin JE (2008). Interleukin-6 and atrial fibrillation in patients with coronary artery disease: data from the Heart and Soul Study. Am Heart J.

[21] Li X, Wu X, Chen X (2023). Selective blockade of interleukin 6 trans-signaling depresses atrial fibrillation. Heart Rhythm.

[22] Jia X, Cheng X, Wu N (2021). Prognostic value of interleukin-6 in atrial fibrillation: a cohort study and meta-analysis. Anatol J Cardiol.

[23] Peng C, Lu Y, Li R (2024). Neuroimmune modulation mediated by IL-6: a potential target for the treatment of ischemia-induced ventricular arrhythmias. Heart Rhythm.

[24] Lazzerini PE, Laghi-Pasini F, Bertolozzi I (2017). Systemic inflammation as a novel QT-prolonging risk factor in patients with torsades de pointes. Heart.

[25] Negreva M, Georgiev S, Vitlianova K, Arabadzhieva D (2015). Interleukin 8: changes in paroxysmal atrial fibrillation. J Adv Med Med Res.

[26] Cozac DA, Somkereki C, Huțanu A, Nicoara TR, Scridon A (2025). The impact of basal inflammatory status on post-CABG atrial and ventricular ectopy and remodeling pathways. Medicina (Kaunas).

[27] Aromolaran AS, Srivastava U, Alí A (2018). Interleukin-6 inhibition of hERG underlies risk for acquired long QT in cardiac and systemic inflammation. PLoS One.

[28] Szabó G, Farkas V, Grunnet M, Mohácsi A, Nánási PP (2011). Enhanced repolarization capacity: new potential antiarrhythmic strategy based on HERG channel activation. Curr Med Chem.

[29] Lazzerini PE, Laghi-Pasini F, Acampa M (2019). Systemic inflammation rapidly induces reversible atrial electrical remodeling: the role of interleukin-6–mediated changes in connexin expression. J Am Heart Assoc.

[30] Hegemann N, Barth L, Döring Y, Voigt N, Grune J (2024). Implications for neutrophils in cardiac arrhythmias. Am J Physiol Heart Circ Physiol.

[31] Jeong EM, Liu M, Sturdy M (2012). Metabolic stress, reactive oxygen species, and arrhythmia. J Mol Cell Cardiol.

[32] Nash SD, Cruickshanks KJ, Klein R (2013). Long-term variability of inflammatory markers and associated factors in a population-based cohort. J Am Geriatr Soc.

[33] Medenwald D, Kors JA, Loppnow H (2014). Inflammation and prolonged QT time: results from the Cardiovascular Disease, Living and Ageing in Halle (CARLA) study. PLoS One.

[34] Marott SC, Nordestgaard BG, Zacho J (2010). Does elevated C-reactive protein increase atrial fibrillation risk? A Mendelian randomization of 47,000 individuals from the general population. J Am Coll Cardiol.

[35] Cao MY, Liu D, Zhang XY, Tian QY, Zhang Q, Wang YX (2022). Association of C-reactive protein with cardiovascular outcomes: a Mendelian randomization study in the Japanese population. Biomed Environ Sci.

[36] Shitole SG, Heckbert SR, Marcus GM (2024). Assessment of inflammatory biomarkers and incident atrial fibrillation in older adults. J Am Heart Assoc.

[37] Gomez SE, Parizo J, Ermakov S (2023). Evaluation of the association between circulating IL-1β and other inflammatory cytokines and incident atrial fibrillation in a cohort of postmenopausal women. Am Heart J.

[38] Zia I, Johnson L, Memarian E, Borné Y, Engström G (2021). Anthropometric measures and the risk of developing atrial fibrillation: a Swedish cohort study. BMC Cardiovasc Disord.

[39] Cinti S, Mitchell G, Barbatelli G (2005). Adipocyte death defines macrophage localization and function in adipose tissue of obese mice and humans. J Lipid Res.

[40] Lee MY, Wang Y, Mak JC, Ip MS (2016). Intermittent hypoxia induces NF-κB-dependent endothelial activation via adipocyte-derived mediators. Am J Physiol Cell Physiol.

[41] Trayhurn P, Wood IS (2004). Adipokines: inflammation and the pleiotropic role of white adipose tissue. Br J Nutr.

[42] Zhang Y, Wang JH, Zhang YY (2016). Deletion of interleukin-6 alleviated interstitial fibrosis in streptozotocin-induced diabetic cardiomyopathy of mice through affecting TGFβ1 and miR-29 pathways. Sci Rep.

[43] Frangogiannis NG (2021). Cardiac fibrosis. Cardiovasc Res.

[44] Sinha F, Schweda F, Maier LS, Wagner S (2023). Impact of impaired kidney function on arrhythmia-promoting cardiac ion channel regulation. Int J Mol Sci.

[45] Potpara TS, Jokic V, Dagres N (2016). Cardiac arrhythmias in patients with chronic kidney disease: implications of renal failure for antiarrhythmic drug therapy. Curr Med Chem.

[46] Shang W, Li L, Huang S (2016). Chronic kidney disease and the risk of new-onset atrial fibrillation: a meta-analysis of prospective cohort studies. PLoS One.

[47] Sarmiento-Cobos M, Valera R, Botero Fonnegra C (2022). Ventricular conduction improvement after pericardial fat reduction triggered by rapid weight loss in subjects with obesity undergoing bariatric surgery. Surg Obes Relat Dis.

[48] Qiu QF, Peng C, Li ZY (2024). [Preliminary study on the role and mechanism of IL-6 receptor antagonists in improving post-infarction ventricular arrhythmia]. Zhonghua Xin Xue Guan Bing Za Zhi.

